# ^18^F-FDG PET-CT for Surveillance of Brazilian Patients with Li-Fraumeni Syndrome

**DOI:** 10.3389/fonc.2015.00038

**Published:** 2015-02-19

**Authors:** Sonia Tereza Santos Nogueira, Eduardo Nóbrega Pereira Lima, Amanda França Nóbrega, Ivone Do Carmo Gonçalves Torres, Marcelo Cavicchioli, Pierre Hainaut, Maria Isabel Waddington Achatz

**Affiliations:** ^1^Oncogenetics, AC Camargo Cancer Center, São Paulo, Brazil; ^2^Nuclear Medicine, AC Camargo Cancer Center, São Paulo, Brazil; ^3^International Prevention Research Institute (iPRI), Lyon, France

**Keywords:** Li-Fraumeni, PET-CT, screening, oncogenetic, p.R337H

## Abstract

**Purpose:** To evaluate the effectiveness of ^18^F-Fluorodeoxyglucose positron emission tomography/computed tomography (^18^F-FDG-PET/CT) for detecting early cancer in carriers of germline *TP53* mutation, the genetic defect underlying Li-Fraumeni and related syndromes, which predisposes to many forms of cancer throughout life.

**Patients and methods:** A total of 30 adult patients from six families with germline *TP53* mutations were recruited. These patients did not have a diagnosis of cancer in the 24 months preceding the study. Anomalous concentrations from whole-body ^18^F-FDG-PET/CT were assessed by two independent experts. Suspicious lesions were excised and subjected to pathological examination.

**Results:** A total of 6/30 patients showed abnormal ^18^F-FDG-concentration. Confirmation studies revealed three cases of cancer, including one lung cancer, one ovarian cancer, and one disseminated breast cancer. Three patients had non-malignant lesions (one Bartholin’s cyst and two cases of reactive lymph nodes).

**Conclusion:** ^18^F-FDG-PET/CT is effective in detecting cancer in subjects who are asymptomatic according to current screening guidelines. These results further suggest that ^18^F-FDG-PET/CT is an appropriate method for surveillance of cancer risk in *TP53* mutation carriers.

## Introduction

Li-Fraumeni syndrome (LFS) is an autosomal-dominant hereditary cancer predisposition syndrome associated with germline mutations in the tumor suppressor gene *TP53* (1, 2). *TP53* mutation carriers have a 50% risk for development of a wide spectrum of malignant tumors by age 30 (3). Several clinical definitions of LFS have been proposed, identifying incomplete forms termed Li-Fraumeni-like (LFL) (4, 5). According to current recommendations (6), referral of a subject for *TP53* mutation testing should be considered if: (i) a proband with a typical LFS/LFL tumor (soft-tissue sarcoma, osteosarcoma, brain tumor, pre-menopausal breast cancer, adrenocortical carcinoma, leukemia, or bronchiolo-alveolar carcinoma) before 46 years with at least one first or second-degree relative with a LFS/LFL tumor before age 56; (ii) a proband with multiple tumors, two of which belong to LFS spectrum and the first one diagnosed before age 46; (iii) a proband with adrenocortical carcinoma or choroid plexus tumor irrespective of family history (7). Germline *TP53* mutations are detected in 29% of the cases fulfilling these criteria (6).

The clinical heterogeneity of LFS raises complex challenges for the management of cancer risk. The guidelines of the National Comprehensive Cancer Network (NCCN) propose a risk management strategy based on clinical examination and imaging with ultrasonography, mammography, and nuclear magnetic resonance imaging (MRI) for breast cancer and colonoscopy for colorectal cancer. Recently, a surveillance protocol based on these principles has shown efficacy in reducing cancer mortality of *TP53* germline mutations carriers (8). This protocol includes additional intensive screening strategies in children such as rapid whole-body MRI, brain MRI, abdominal ultrasound examination, and biochemical test for markers of adrenal cortical function (8). However, this strategy addresses only some of the many cancer types that may occur in LFS families. Thus, alternative screening methods are needed, that may detect a larger spectrum of LFS cancers. A pilot study of screening with ^18^F-Fluorodeoxyglucose positron emission tomography/computed tomography (^18^F-FDG-PET/CT) has been reported in 15 adult carrying germline *TP53* mutations (9). This study detected three prevalent cases of malignant lesions (20%), suggesting that ^18^F-FDG-PET/CT may help in improving surveillance of cancer risk in LFS/LFL.

In the present study, we describe the use ^18^F-FDG-PET/CT for cancer detection in 30 clinically asymptomatic *TP53* mutation carriers undergoing continuous follow-up according to NCCN guidelines (2009 version) and with no diagnosis of cancer within 2 years preceding the study. The study was conducted in a population of patients from Southern Brazil, where LFS is particularly common due to a founder *TP53* mutation, p.R337H (7).

## Materials and Methods

### Patients and selection criteria

Institutional Review Board approval was obtained and all patients included in this study provided informed consent. Thirty patients who carried a *TP53* germline mutation were selected at the Department of Oncogenetics, Hospital A.C. Camargo, São Paulo, Brazil. Individuals who fulfilled criteria for *TP53* mutation testing were included. Upon positive testing, a total of 30 subjects were consecutively recruited. All patients matched clinical criteria for LFS or LFL (Table 1) and were not diagnosed with cancer within 2 years preceding inclusion in the study. Exclusion criteria were age under 18, refusal to participate in the study, and history of metastatic cancer. The study was carried out from March 2009 to March 2011.

**Table 1:** **Families, mutations, and diagnosis criteria for patients recruited for ^18^F-FDG PET-CT imaging**.

Family	Mutation	Number of patients	Diagnosis criteria^a^	cDNA position
Y12	p.R337H	23	LFS	c.1010G > A
Y27	p.R337H	1	LFL(B)	c.1010G > A
Y99	p.R337H	2	LFL(C)	c.1010G > A
Y97	p.R337H	1	LFS	c.1010G > A
Y79	p.T125T	2	LFL(C)	c.375G > A
Y131	IV8 + G > A	1	LFS	(Intronic)

*^a^Families were classified according to the clinical definitions of Li-Fraumeni syndrome (LFS, proband with sarcoma at 45 years and a first-degree relative with tumor at 45 and another close relative with tumor at 45, or sarcoma at any age) or Li-Fraumeni-like syndromes; LFL(B), proband with any childhood cancer or sarcoma, brain tumor or adrenocortical carcinoma at 45 years, with one first- or second-degree relative with typical LFS cancer, including sarcoma, breast cancer, brain tumor, leukemia, or adrenocortical carcinoma at any age, plus one first- or second-degree relative in the same lineage with any cancer diagnosed under age 60; LFL(C), proband with tumor belonging to LFS tumor spectrum (soft-tissue sarcoma, osteosarcoma, brain tumor, pre-menopausal breast cancer, adrenocortical carcinoma, leukemia, lung bronchoalveolar cancer) before age 46 years and at least one first- or second-degree relative with LFS tumor (except breast cancer if proband has breast cancer) before age 56 years or with multiple tumors; or proband with multiple tumors (except multiple breast tumors), two of which belong to LFS tumor spectrum and first of which occurred before age 46 years; or patient with adrenocortical carcinoma or choroid plexus tumor, irrespective of family history*.

### ^18^F-Fluorodeoxyglucose positron emission tomography/computed tomography

^18^F-FDG-PET/CT whole-body scans were performed at the Nuclear Medicine division of the Imaging Department, Hospital A.C. Camargo, with PET-CT GEMINI Philips equipment. Patients were fasting for 6 h before undergoing examination. Venous injection of 5.2 MBq/Kg of ^18^F-FDG was performed after a 30-min rest and blood glucose level verification. The examination protocol started with no contrast CT and was followed by the PET acquisition, from the head up to the medium third of the thigh 90 min after injection. Images were analyzed and interpreted by one of three nuclear physicians and confirmed by a second. The criteria used for evaluation of the examinations were the existence of visually abnormal sites of ^18^F-FDG concentration and the quantitative measurement of the standardized uptake value (SUV).

## Results

A total of 30 patients were included (18 women, 12 men; mean age: 43 ± 16.5 years; age range, 19–76 years), representing a total of six families with a diagnosis of LFS or LFL syndrome. The Brazilian founder p.R337H *TP53* mutation was present in 90% of the patients (27/30, belonging to four apparently unrelated families). Prior to testing for *TP53*, all patients were already under surveillance according to NCCN guidelines (10), based on their familial history. None of them showed signs of cancer disease.

**Figure 1:**
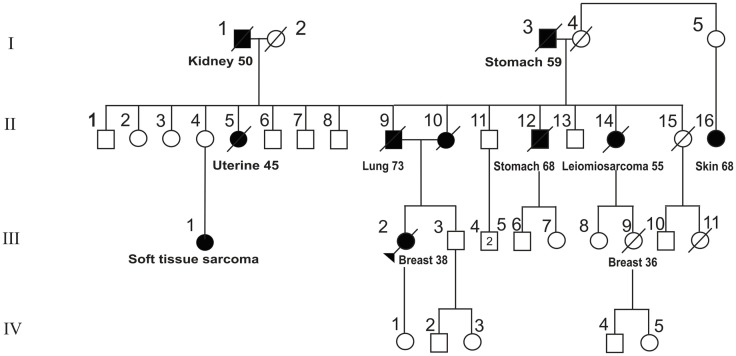
**Pedigree of family Y0029**. The index patient is indicated by an arrow. Full boxes/circles: patient affected by a malignant tumor. Age of onset and tumor type are given below each patient’s symbol.

**Figure 2:**
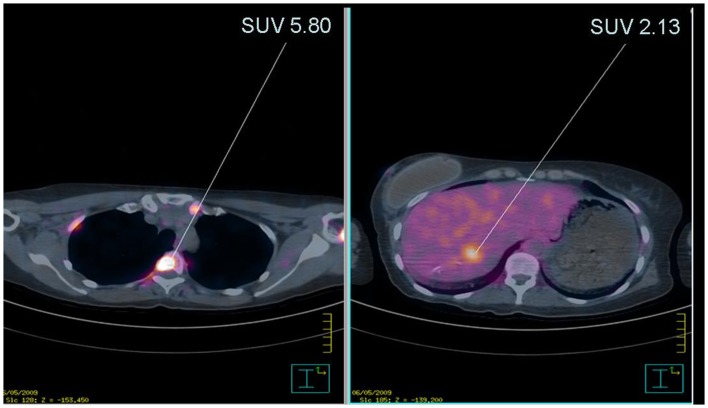
**Anomalous concentration of ^18^F-FDG in index patient of family Y0029**. Left panel: anomalous signal in bone projections of bone corresponding to predominantly intramedullary lesions. The arrow points to a lesion with SUV 5.80. Right panel: anomalous signal in the liver. The arrow points to a lesion with SUV 2.13.

Sites with abnormal concentration of ^18^F-FDG were observed in 20% (6/30) of the patients (Table 2). Among the six patients, findings were further assessed using conventional imaging and/or histopathology. One lesion was identified in a pelvic location as Bartholin’s cyst and patient was returned to clinical follow-up according to NCCN guidelines (10). The second case was a woman aged 40 from a family with the mutation p.R337H (shown in Figure 1, where the patient is identified by an arrow). This patient had previous history of breast cancer at age 38. ^18^F-FDG-PET/CT images were suggestive of diffuse bone (intramedullary) and liver dissemination of an invasive ductal breast carcinoma (Figure 2). The lesions were not assessed by pathological examination (PE). Of note, this patient underwent chest CT and whole-body bone scintillography 1 month before ^18^F-FDG-PET/CT, with no signs of metastasis.

**Table 2:** **Characteristics of patients with positive FDG-PET/CT findings**.

Patient number	Gender/age	Previous history of cancer (age)	Concentration area	*TP53* mutation	SUV max	Histopathological or imaging diagnosis
04	F/67	Breast (51, 61); thyroid (62); skin (65)	Lung	p.R337H	1.7	Mixed acinar/bronchiolo-alveolar carcinoma
11	F/62	No	Pelvis	p.T125T	5.1	Adenocarcinoma of the ovary
07	F/40	Breast (38)	Bones/liver	p.R337H	5.8	Metastases of breast cancer
01	F/29	No	Pelvis	p.R337H	2.02	Bartholin cyst
16	F/60	No	Mesenteric root	p.T125T	7.0	Lymph nodes; reactive hyperplasia
20	M/56	Thyroid (54)	Right axillary	p.R337H	2.0	Single lymph node; reactive hyperplasia

*F, female; M, male*.

**Figure 3:**
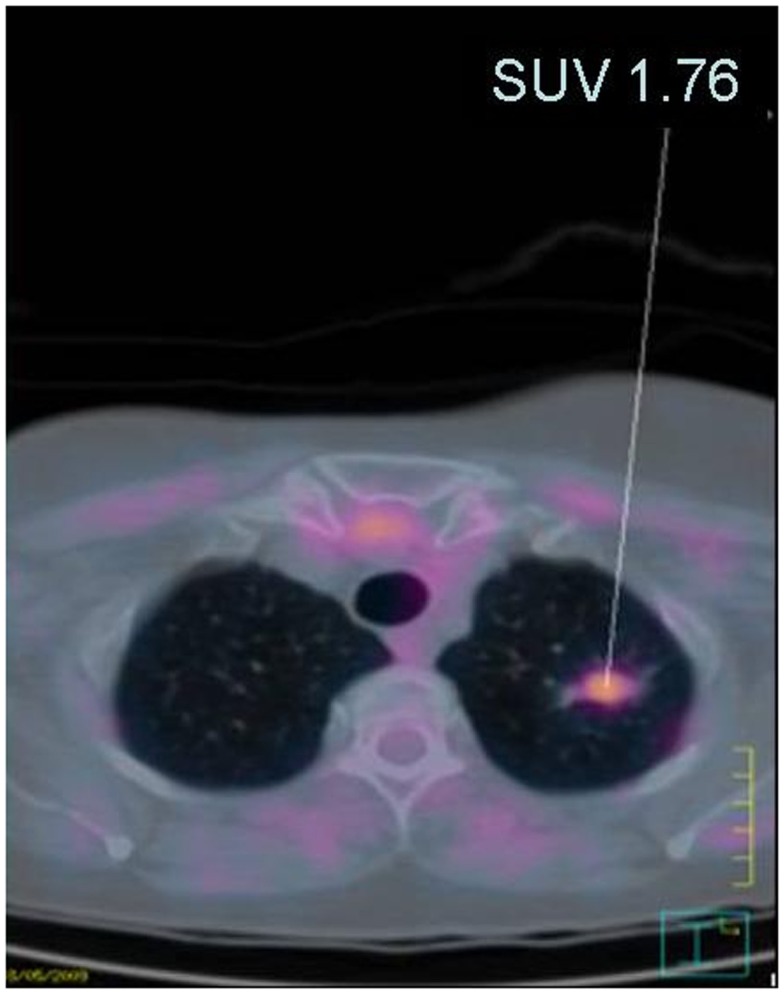
**Image fusion of PET with CT showing anomalous concentration of ^18^F-FDG**. A lesion localized in the apical pulmonary region is shown (SUV 1.76).

Lesions in the four remaining patients were submitted to biopsies. In two patients, lesions were confirmed as malignant tumors by PE (Table 2). An apical left lung lesion was detected in a woman aged 67 with a history of bilateral breast cancer at 51 and 61 years, thyroid cancer at 62, and non-melanoma skin cancer at 65, PE revealed a mixed invasive adenocarcinoma with acinar and lepidic histological features. The patient was staged as IIIB (Figure 3). She underwent standard chemotherapy and was in remission 1 year after diagnosis. She relapsed 24 months post-diagnosis, with development of brain metastases. Another patient aged 62 years with no previous history of malignant disease presented with concentrations of ^18^F-FDG in the abdominal and pelvic area that identified a pelvic mass, peritoneal carcinomatosis, and multiple peri-bowel and peri-hepatic implants. PE confirmed the diagnosis of undifferentiated carcinoma with probable ovarian origin, stage IV. Among the two remaining patients, one presented with a remarkably high ^18^F-FDG concentration at the mesenteric root and the other showed an axillary lesion. In both cases, biopsies revealed a non-malignant hyperplasia of lymph nodes, of probable reactive origin.

The 27 patients with negative ^18^F-FDG-PET/CT status or with non-malignant findings were returned to surveillance according to NCCN guidelines. In summary, ^18^F-FDG-PET/CT imaging detected malignant lesions in 3 of 30 patients who were non-symptomatic in screening according to NCCN guidelines.

## Discussion

The surveillance of patients with germline *TP53* mutations is complex due to the wide range of cancer types related to LFS, as well as to lifelong predisposition to cancer. The NCCN guidelines for patient surveillance recommend follow-up using a combination of clinical examination, breast imaging, and colonoscopy (10). However, there is concern that this panoply is insufficient to capture at an early stage the wide range of malignant lesions these patients may develop.

In the present study, we have used ^18^F-FDG-PET/CT to monitor the status of 30 germline *TP53* mutation carriers from six independent families who met at least one of the diagnosis criteria of LFS/LFL. We found anomalous ^18^F-FDG concentration in 6/30 subjects with germline *TP53* mutation (20%). Further investigation confirmed malignant lesions in 3/30 subjects (10%). Our results suggest that ^18^F-FDG-PET/CT is an appropriate means for surveillance of cancer risk in *TP53* mutation carriers.

All patients were included in routine LFS/LFL follow-up according to NCCN guidelines and did not show evidence of cancer at the time of enrollment in the study. Our results are compatible with those of the previous study by Masciari et al. (9), who analyzed by ^18^F-FDG-PET/CT a group of 15 *TP53* mutation carriers (or obligate carriers) with no diagnosis of cancer within 5 years prior to recruitment. They found malignant lesions in three of them (20%) (two cases of thyroid cancer, stages II and III, and one case of adenocarcinoma of the esophagus, stage II). These authors also detected areas of abnormal ^18^F-FDG concentration in five patients, who were found to correspond to non-malignant lesions. Thus, together with these previous results, our study provides evidence for the usefulness of ^18^F-FDG-PET/CT for cancer surveillance in LFS/LFL.

The analysis of cumulative rates of occurrence of cancers in *TP53* mutation carriers compiled in the IARC *TP53* database (http://www-p53.iarc.fr) (11) suggests that, in adulthood, between 1 and 2% of mutation carrier develop cancer by each year of age. Our results and those of Masciari et al. (9) identified that 10 and 20%, respectively, of the subjects had a prevalent cancer. This observation suggests that malignant lesions in *TP53* mutation carriers may occur at a higher rate than expected on the basis of the data compiled in the IARC database. Further studies on the risk of cancer per year of age in *TP53* mutation carriers are needed to understand this discrepancy.

It is striking to note that the malignant lesions detected in our study were all at advanced stage. Thus, this observation raises the question of whether ^18^F-FDG-PET/CT is adequate for detection of early lesions in *TP53* mutation carriers. Moreover, it suggests that, in order to be effective, ^18^F-FDG-PET/CT should be repeated at relatively short time intervals of approximately 1 year. Such a strategy would raise a number of questions with respect to cost-effectiveness and, in particular, to the need of limiting exposure to ionizing radiation in patients with germline *TP53* mutations (12). Common practice discourages the use of radiation in LFS/LFL subjects because they are at risk for the development of second malignancies (13). To date, there is no evidence that ^18^F-FDG-PET/CT levels of radiation may represent an additional risk factor in *TP53* germline mutation carriers. However, this question deserves careful, further assessment of the exact nature and level of the risk.

Several groups have begun to utilize an intensive screening strategy including rapid whole-body MRI, that may not be available at many centers. Prospective studies are needed to demonstrate the effectiveness of this protocol in adults and children with LFS.

In conclusion, our results add to previous data suggesting that ^18^F-FDG-PET/CT may be an appropriate means for surveillance of cancer risk in *TP53* mutation carriers. They also suggest that the use of ^18^F-FDG-PET/CT in research programs is warranted to gain a better understanding of the risk of developing cancer and of the actual rate of cancer development in carriers of *TP53* mutations. Further studies are needed to assess the conditions of use of ^18^F-FDG-PET/CT in LFS/LFL surveillance strategies and compare with other whole body modalities as whole body enhanced CT and whole body MR with diffusion.

## Conflict of Interest Statement

The authors declare that the research was conducted in the absence of any commercial or financial relationships that could be construed as a potential conflict of interest.
